# Automated underwater image analysis reveals sediment patterns and megafauna distribution in the tropical Atlantic

**DOI:** 10.1038/s41598-025-12723-y

**Published:** 2025-07-28

**Authors:** Benson Mbani, Jens Greinert

**Affiliations:** 1 DeepSea Monitoring Group, GEOMAR Helmholtz Center for Ocean Research Kiel, Wischhofstraße 1-3, 24148 Kiel, Germany; 2 Institute of Geosciences, Kiel University, Ludewig-Meyn-Str. 10-12, 24118 Kiel, Germany

**Keywords:** Ocean sciences, Computer science

## Abstract

The deep-sea comprises diverse habitats and species whose characterisation provides crucial insights into the health and resilience of our oceans. Whereas direct sampling enables investigation of the vertical variability of the seafloor at small spatial scales, optical imaging allows for multi-scale assessment of the spatial distribution of (mega)benthos and substrates. However, modern seafloor imaging surveys typically generate thousands of images that are infeasible to manual annotation. Consequently, transforming these terabyte-scale datasets into actionable insights requires automated workflows. Here, we deployed two A.I workflows to automate the annotation of substrates and megafaunal taxa in seafloor images from the tropical North Atlantic. Clustering, feature space visualisation and multivariate statistical analysis techniques were used to classify the seafloor into habitats, estimate megafaunal distribution patterns, and to identify environmental drivers that influence observed patterns. We found that the seabed here formed seven clearly distinct clusters, with visible sub-partitions observed in each cluster. Investigations revealed a gradient of sediment disturbance due to biogenic activity, with images showing little-to-no sediment disturbance mapping to one half of the feature space, whereas images exhibiting visibly vigorous sediment reworking mapping to the other half of the feature space. Also, megafaunal abundances were 14 times higher in the shallower Eastern region of the seabed, potentially due to higher Particulate Organic Carbon flux and relatively warmer temperatures. Moreover, geographic clustering of megafauna was observed in topographically complex features such as slopes of submarine canyons and on top of seamounts, where heterogeneity created diverse microhabitats and unique niches that megafauna could exploit.

## Introduction

The deep sea comprises a wide range of benthic habitats, is home to diverse sets of floral and faunal communities, and is also the largest biome on earth^1^. Despite this, the biodiversity within these remote ecosystems is still largely under-sampled^2^ and patchily documented^3^even after accounting for the increased frequency of scientific expeditions over the past decades^4^. This is because of logistical, technological and financial challenges that constrain the overall spatio-temporal extents that can be reasonably investigated^5^. Besides, in-situ and/or visual characterization of organisms in the deep sea can sometimes be non-trivial, either because organisms in these environments are new to science, or because their distribution patterns (and ecosystem processes) are not yet properly understood^6^. Recent scientific studies have also provided conclusive evidence showing a global decline in marine biodiversity as a result of both natural and anthropogenic factors e.g. overfishing, pollution, coastal development, natural climate variability, and long-term geological processes like sedimentation and tectonic/hydrothermal activities^7^. To better quantify and address this biodiversity decline, globally coordinated efforts are required to not only increase the frequency and spatial extent of marine ecosystem surveys, but also to expedite the analysis of the acquired datasets. These datasets include high resolution images and videos collected using platforms such as Autonomous Underwater Vehicles (AUVs), Remotely Operated Vehicles (ROVs), and towed Ocean Floor Observation Systems (OFOS)^8^. While imaging sensors attached onto these platforms conveniently allow for non-invasive surveying of deep-sea environments in high resolution, they generate huge volumes of imagery for which manual interpretation is unfeasible^9^. As a result, automated workflows based on emerging digital technologies are required to expedite the processing and annotation of images, thereby providing comprehensive baseline information on geological, sedimentological and biological properties of marine ecosystems^10^.

Modern machine learning techniques have demonstrated the capacity for rather quick yet accurate extraction of semantic information from large sequences of image and video datasets^11^. In particular, pre-trained computer vision models based on convolutional neural networks are nowadays readily available for download from open-source repositories (e.g. TensorFlow Hub and PyTorch Hub), and can be directly deployed as-is to accomplish common tasks such as image enhancement, classification, object detection and dense pixel segmentation^12^. Given that most of these pre-trained models were originally trained to identify common objects on terrestrial images using benchmark datasets like ImageNet^13^, the models require fine-tuning using annotated underwater images before they can be useful for applications such as marine habitat mapping and biodiversity assessment^14^. This requirement poses significant bottlenecks in at least two dimensions: First, annotating images after every scientific expedition is costly, unscalable and therefore undesirable; Second, marine environments naturally exhibit low density of megafauna with increasing depth, which implies that organisms will be visible on only a handful (out of possibly tens of thousands) of acquired images that are typically unknown apriori^15^. Addressing these challenges requires automated A.I-based seafloor classification and megafaunal detection workflows that not only work well in a specific working area, but that are easily generalizable to other marine ecosystems^16^. Such a generalised approach saves human analysts the trouble of annotating datasets from scratch, allowing them to concentrate on refining and assigning semantic morphospecies labels only to auto-generated annotations^9^. The semantic annotations can then form the basis of downstream assessment of spatio-ecological distribution patterns of habitats, megafauna and environmental drivers.

Megafaunal species are not distributed randomly in space^17^. Instead, they cluster together into biotically-similar communities that are in turn structured by processes and variables such as bathymetric gradients, geomorphology, food availability, chemical/physical bottom water conditions, as well as sediment or hardground properties related to settling, hiding or breeding^18^. Characterization of these megafaunal patterns is typically performed using multivariate statistical analysis techniques^19^, which we also found to be applicable in this present study given that our image-derived annotations comprised abundances for multiple taxa. Before using these statistical techniques to assess the distribution of megafauna, however, it is necessary to first account for the inconsistent visual footprints among respective images due to their variable acquisition heights^16^. This inconsistency can be resolved by systematically defining standardised sampling units (e.g. equal-area quadrats or fixed-length linear transects), within which megafauna counts are pooled and normalised relative to the actual observed area^20^. Collectively, these sampling units encode biotic information as abundances that can simply be binned and plotted on a choropleth map to visualise spatial distribution of megafauna. Alternatively, ordination techniques such as non-metric multidimensional scaling (nm-MDS) can be used to graphically display inter-relationships among the different taxa in feature space^19^. Furthermore, an arbitrary number of relevant environmental variables can also be superimposed on the ordination plot, allowing for a more nuanced visual assessment of the (subset of) abiotic factors that influence the different clusters of megafauna^21^. Finally, spatial autocorrelation analysis may also be used to reveal megafaunal hotspots, cold spots and outliers^22^.

Past studies have proposed various workflows and approaches for semi-automating the annotation of underwater images. Supervised approaches have been used extensively for tasks such as image-based seafloor classification because they are capable of generating accurate annotations (in inference mode) whenever sufficient number of labelled examples are available for training^23^. To facilitate rapid innovation, experimentation, reproducibility and evaluation of supervised models, there have been studies aimed at curating standardised (labelled) benchmark datasets from both real^24^ and simulated marine environments^25^. Whereas classical machine learning techniques such as random forests^26^ and support vector machines^27^ were predominantly incorporated in marine image analysis workflows in the past decade, recent studies almost exclusively use convolution neural networks^28^. In particular, models such as YOLO^29^, RetinaNet^30^ and Faster R-CNN^31^ are now widely used for detecting, localising and classifying flora and fauna from images and videos after training with just hundreds of training examples per class^11^. There is also evidence that these deep learning models are computationally resource-intensive only during model training, otherwise the models are remarkably efficient when making predictions in inference mode^32^. Unsupervised approaches such as template matching^33^ and superpixel-based segmentation have also been used in previous studies^34^typically as an initial preliminary step e.g. to cheaply generate weak annotations^35^or to quickly sort images based on natural groupings^36^. Some studies still rely exclusively on human workforce to exhaustively annotate their datasets, which is accurate (and *arguably* the gold standard) but also very costly and non-scalable^37^. Regardless of the chosen annotation strategy, the generated annotations are normally used as inputs to downstream spatio-ecological workflows that rely on e.g. multivariate statistics and measures of spatial autocorrelation to characterise abundances, diversity, and spatial distribution patterns of megafauna ^19,17,21^.

**Fig. 1:**
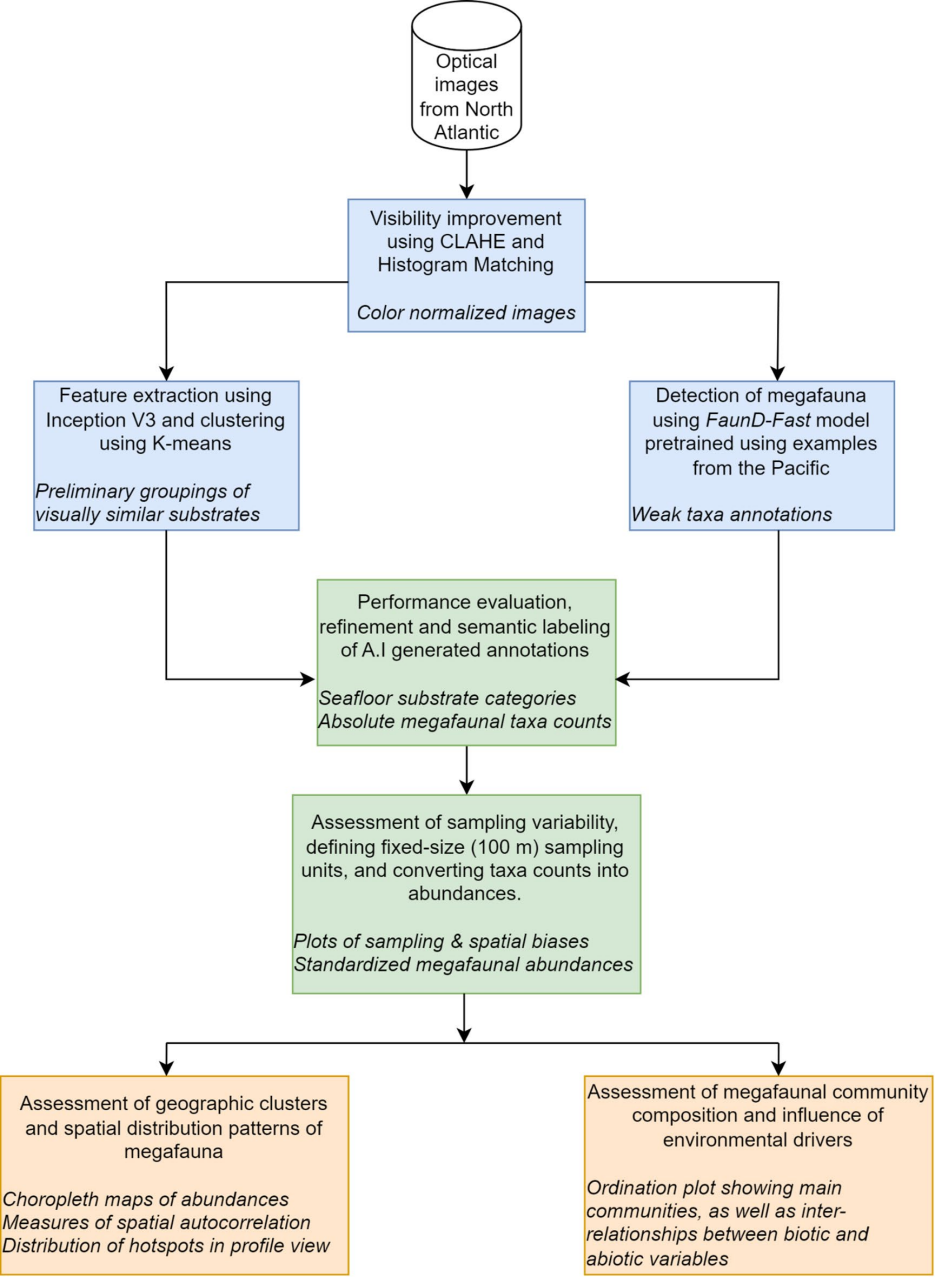
Flow diagram showing the interconnected components of our proposed workflow that comprises three key steps: First, we enhance the visibility of images before deploying two A.I workflows to classify seafloor images into habitat classes, and also to detect megafaunal taxa; Second, we inspect and assign semantic taxa labels to the auto-generated *weak* annotations, before converting the absolute taxa counts into abundances relative to actual observed area within our predefined fixed-size sampling units; Finally, we use the abundances to characterize spatial distribution patterns of megafauna, and also to graphically display interrelationships among biotic and abiotic variables in ordination feature space.

Here, we investigated seafloor habitats and benthic megafaunal distribution patterns in the tropical North Atlantic using the conceptual workflow in Fig. 1. Specifically, we fine-tuned A.I workflows that we previously developed for classifying seafloor habitats^9^ and detecting benthic megafauna^23^ in the Clarion-Clipperton Zone. We used the fine-tuned models to expedite the annotation of a new dataset comprising seafloor images from the tropical North Atlantic. Given that the A.I workflows were originally used for benthic assessments in the Pacific, one broad objective for this study was to investigate the generalizability of the two workflows when presented with dataset from a completely different area and geological setting. Specific objectives were: (1) to reveal subtle variability in seafloor habitat classes using unsupervised machine learning techniques; (2) to semi-automate the detection, localisation and classification of megabenthic taxa from sequences of high-resolution images; (3) to characterise the spatial distribution patterns of the annotated megafauna; (4) to estimate megafaunal abundance, diversity and community composition; and finally, (5) to assess the influence of environmental drivers on the observed distribution patterns.

## Results

This section presents findings from our unsupervised seafloor classification, along with a description of megafaunal taxa that we detected in the area. We further describe the spatial distribution patterns of these megafauna, as well as environmental drivers that influence their distribution in both Eastern and Western regions.

### Visibility improvement

**Fig. 2:**
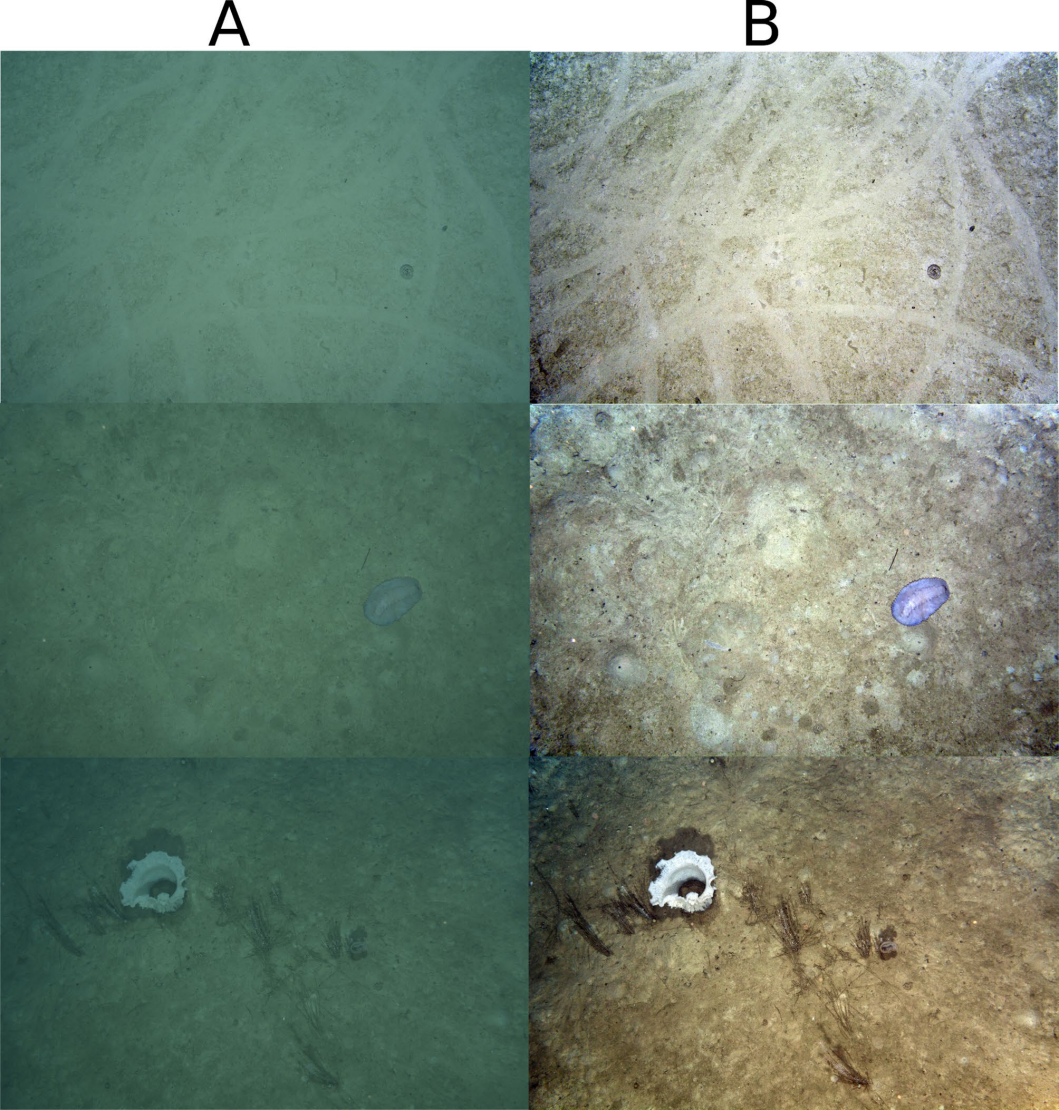
Examples showing visibility improvement transformation from (A) original images, to (B) color normalized images. The megafaunal taxa, animal trails and bioturbation-driven sediment disturbance are now clearly visible in the transformed images.

Qualitative results of our colour correction workflow are shown in Fig. 2. The reduction in image intensity towards the edges is now accounted for, and the overall contrast is enhanced in the transformed image. The correction also removes the greenish haze that was prevalent in the raw images, resulting in good distribution of colours over the entire enhanced image. Collectively, these transformations produce well-illuminated scenes that reveal biota and substrate characteristics with clear contrast e.g. highlighting animal tracks and sediment disturbance due to biogenic activities.

### Seafloor substrate classification

**Fig. 3:**
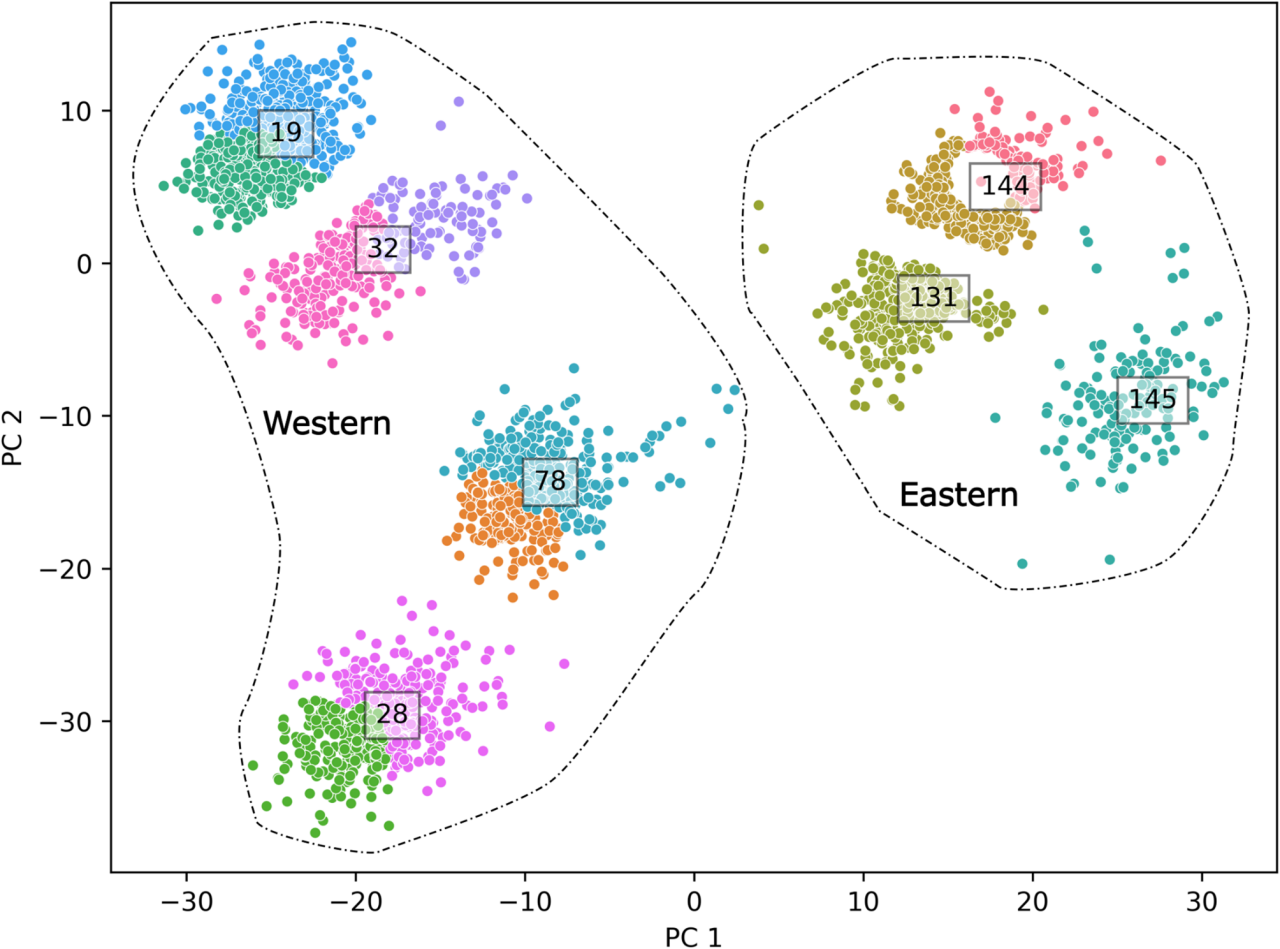
Projection of images (as points) in feature space, color coded by one of 12 seafloor substrate classes. Images from respective dives cluster together because they represent the same geographic region on the seabed and thus have most similar sedimentological and benthic properties. The images also show sub-partitions within dives, which is an indication of subtle differences in seabed substrates at small spatial scales. Overall, there is a clear variability for PC1 that links to increasing intensity of sediment disturbance by bioturbating organisms (higher PC1 values).

Results of our unsupervised seafloor classification are shown in Fig. 3. The classification is based on the extracted visual information for the entire image, which encodes both biogenic and abiogenic properties of the photographed seabed (e.g. bioturbation, lebensspuren, burrows, seafloor morphology, sediment colour, etcetera). Each point corresponds to an individual image mapped in feature space, while colour coding is based on the twelve seafloor classes assigned to the respective images using unsupervised K-means classification.

Sediment sampling during cruise M182 showed that the seafloor is composed of soft sediment of different grain size and composition. Towards the East and in closer proximity to land, the amount of fine grained (silt) terrigenous sediment increases, while towards the West, sediments are strongly dominated by foraminifera shells. The clustering shows that the seabed exhibits subtle differences at small range along a survey transect, while there were clear differences at regional scale separating the different surveys from each other.

By manually inspecting subsample images from each survey-cluster (Supplementary Figure S1), we observed that these differences reflected the extent of sediment disturbance by biogenic activities (on feeding and moving tracks), and in the sediment (burrow holes, sediment mounds). The disturbance was mostly pronounced in the texture of images from the shallower Eastern region (surveys 131, 144, 145), characterised by burrows, pits and rosette-like structures resembling a sweeping polychaete arm. The feature space projection captures this difference, by showing a left-to-right (West to East) gradient in terms of bioturbation intensity.

### Megafaunal abundance and diversity

**Fig. 4:**
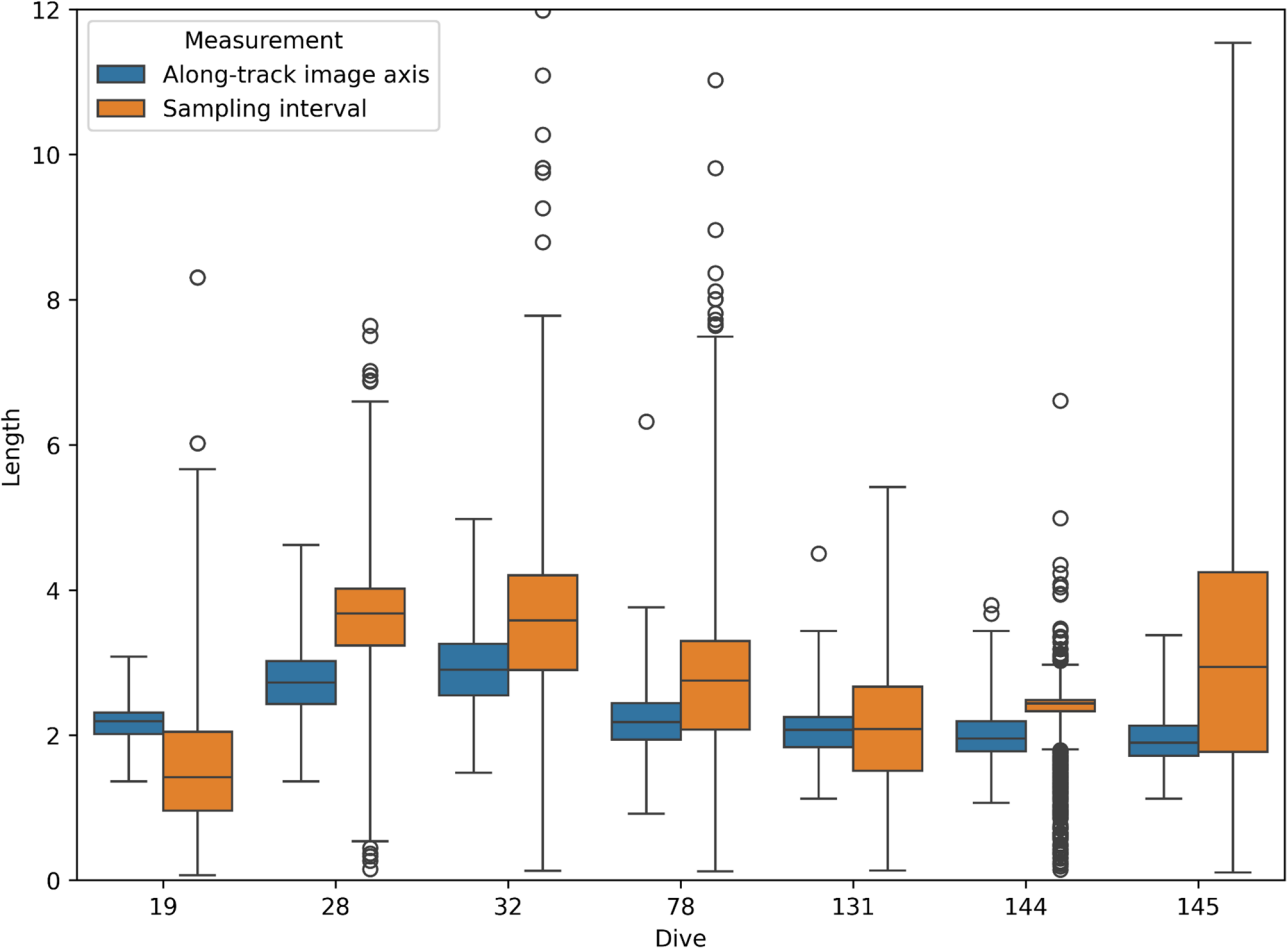
Relationship between average distance between successive images and the average image length in the direction of image acquisition. For a given dive, there was overlap if the image length was shorter than the distance between images.

Our *FaunD-Fast* model detected 10,189 megafaunal organisms belonging to 13 taxa groups. To check for potential double counting of megafauna due to overlapping images, we compared the average distance between successive images against the average length of the along-track image axis (oriented in the direction of image acquisition). The results of this comparison are shown in Fig. 4, where we only found overlap in dive 19 out of the seven dives. Note that dive 19 was also where the sampling frequency was highest (0.2 Hz).

Considering that the remaining six dives did not have overlap, our abundance estimates are still reliable despite the potential double counting in the one dive. In any case, double counting inflates absolute taxa counts but does not affect abundances, since abundances are obtained by dividing absolute counts by the total observed area (which would also be doubled).

**Fig. 5:**
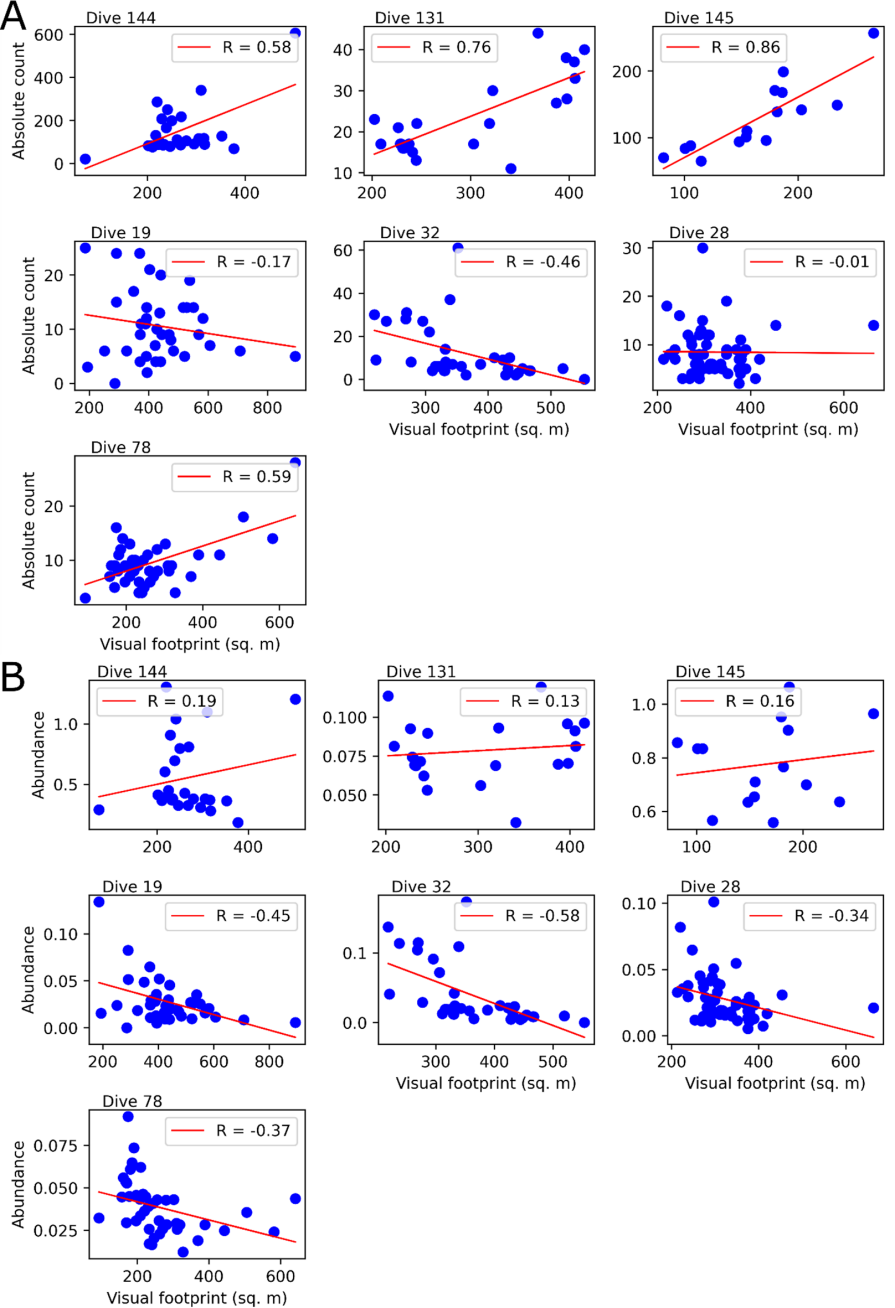
Correlation between visual footprint on the seafloor and (A) absolute megafauna counts, (B) megafaunal abundance. It is clear that images-level annotations are not directly comparable because the number of observed megafauna depends on the seafloor area represented by the image. In contrast, abundances are normalised relative to actual observed area (within standardised sampling units) and are therefore comparable.

Our conversion of absolute megafaunal counts to abundances involved pooling the annotations into 232-hundred-metre-long sampling units, resulting in an average of 38 ± 15 images each. The effect of this conversion to abundances is shown in Fig. 5, where dives in the shallower Eastern region (including dive 78 in the Western region) clearly exhibit positive linear correlations between absolute taxa counts and observed visual footprint (Fig. 5A). This linear relationship is undesirable because it implies that (a) image-level annotations are not directly comparable since observed megafauna counts depend on the arbitrary size of the observed seafloor area rather than natural spatio-ecological processes, and (b) the spatial scale of the observed distribution patterns is ambiguous. In contrast, Fig. 5B shows that our standardisation approach decorrelates the above-described linear relationships, which means that our abundance estimates (at the resolution of the sampling unit) are now directly comparable and usable in downstream ecological analysis.

In terms of proportions, Supplementary Figure S2 shows that the most abundant taxa were Foraminifera (44.95%), Echinodermata (16.66%) and Lebensspuren (14.79%). The proportions of Porifera (6.41%) and Arthropoda (5.20%) were also relatively high, whereas Cnidaria, Sponge-Skeleton, Mollusca, Chordata, Annelida, Ctenophora and Chaetognatha all accounted for less than 5% of the absolute taxa count. We have cropped out examples image patches for each of these taxa groups and provided them in Supplementary Figure S3.

Supplementary Figure S4 further reveals contrasting regional differences in megafaunal abundances between the Eastern and Western regions. Sampling units in the Eastern region recorded higher abundances on average (0.44) compared to those on the Western side (0.03). As would be expected, the shallower Eastern region also recorded significantly high variability in megafaunal abundances (standard deviation = ± 0.35) when compared to the Western region (standard deviation = ± 0.02). Note that high standard deviation (or variability) in megafaunal abundances among sampling units is one of the indicators of ecological heterogeneity.

The observed regional differences in megafaunal abundance were also statistically significant. This is evident from our ANOSIM results (*R* = 0.37; *p* < 0.001) that led us to reject our previously defined null hypothesis *H0: there is no significant difference in biotic composition between the main megafaunal communities.* In addition, results from SIMPER analysis revealed that 50% of the dissimilarity in biotic composition between the two regions could primarily be attributed to four taxa: Porifera (14.46%), Lebensspuren (13.27%), Cnidaria (11.97%) and Mollusca (9.65%). Note that since the depth difference between the Eastern and Western region is approximately 700 m, the above findings also simultaneously describe the two regions in terms of bathymetric differences.

### Spatial distribution of megafauna

**Fig. 6:**
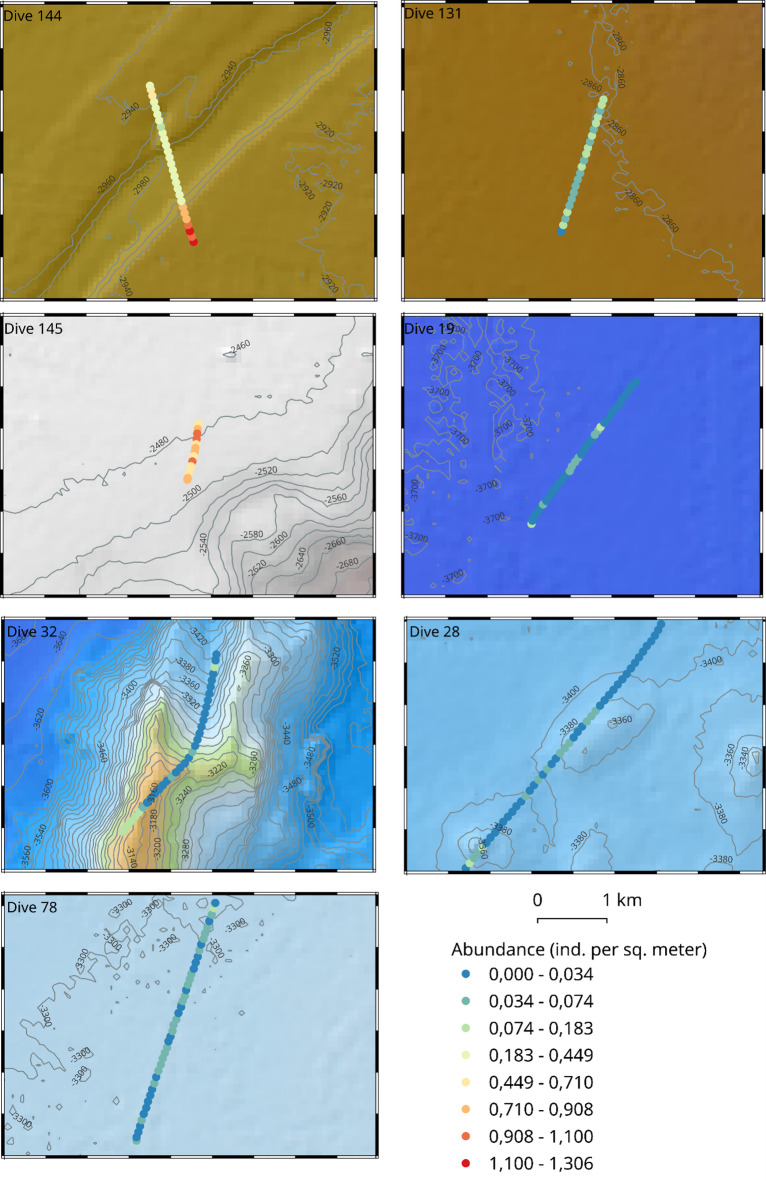
Choropleth maps showing the distribution of megafaunal abundances along respective survey transects. Overall, the dives in the shallow eastern region exhibit high abundance consistently along transects whereas the abundances are low in the deeper Western region, except in topographically complex habitats like on top of seamounts.

The spatial distribution of megafauna along the observational tracks exhibits patterns of geographic clustering at both regional and local scale (Fig. 6). The seabed in the shallower Eastern region (dives 131, 144, 145) was characterised by relatively high abundances throughout the entire transects, except in a few areas on the relatively flat terrain of dive 131. Abundances were significantly higher in topographically complex areas such as on the sides of the submarine canyon (dive 144) as well as the top of the seamount in dive 145. On the other hand, the deeper Western region generally exhibited low megafaunal abundances, with localised areas of higher abundances in complex topographic features such as the top of seamount in dive 32 and the pair of abyssal hills in dive 28.

Maps showing the distribution of each megafaunal taxa along respective deployment tracks are provided in Supplementary Figure S5.

**Fig. 7:**
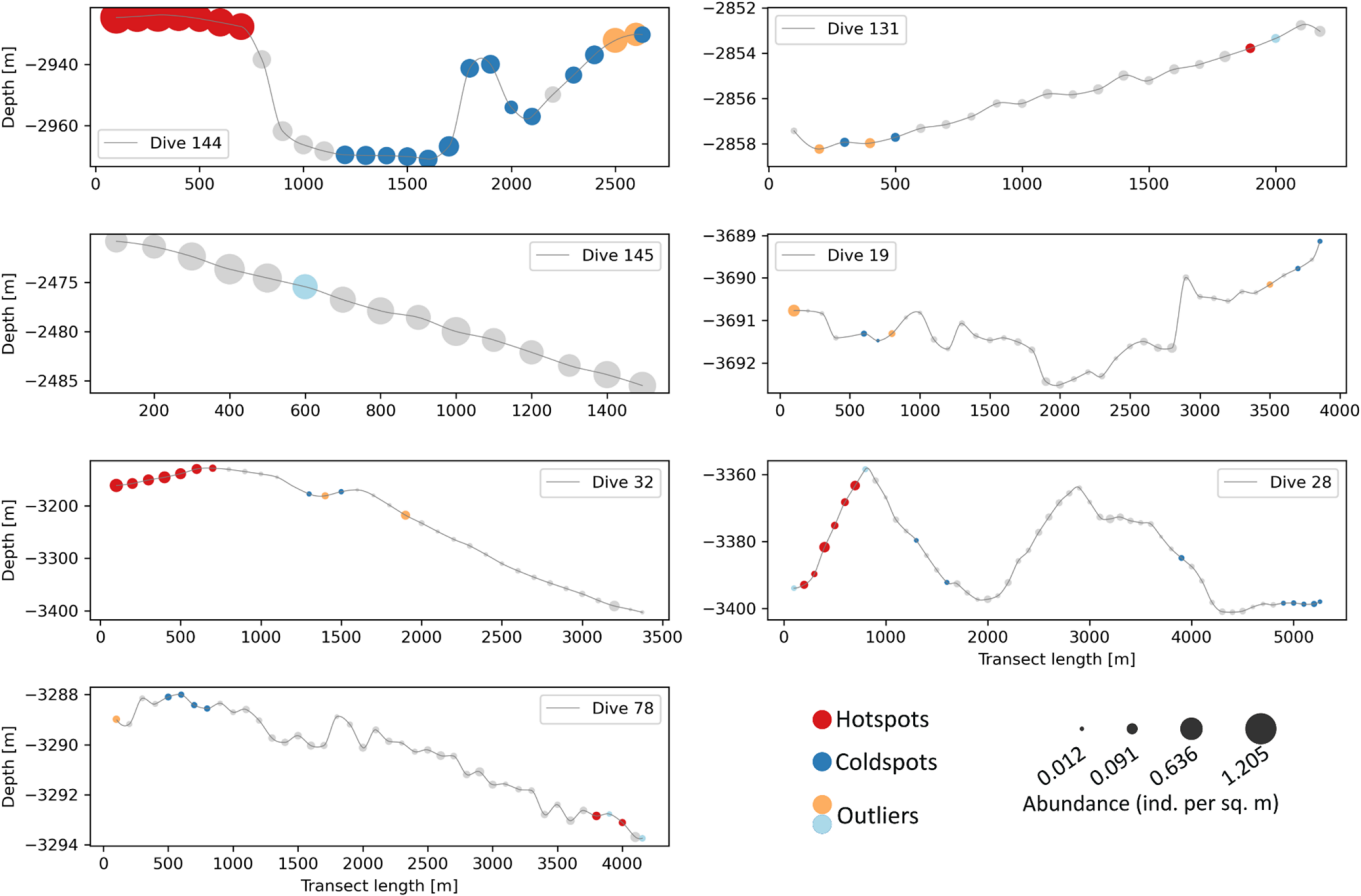
Profile view of geographic clustering showing the distribution of statistically significant hotspot, cold spots and outliers along respective transects. The size of the symbol is proportional to the megafaunal abundance in the corresponding sampling unit at that location. At our chosen scale of analysis (100 m) and small neighborhood size (of 6), the figure shows that hotspots of megafauna are predominantly found in complex topographic features e.g. the top of seamount (dive 32), abyssal hills (dive 28) and on the sides of a submarine canyon (dive 144).

Profile views of hotspots, coldspots and outliers are shown graphically in Fig. 7. Compared to the choropleth map above, only locations with statistically significant clusters of high/low megafaunal abundances (relative to local neighbourhoods) are colour coded.

The Eastern region was characterised by a statistically significant hotspot of high megafaunal abundances at the start of the steep side of the submarine canyon in dive 144, with the other half of the cross section exhibiting coldspots of relatively lower abundances. Contrary to expectations, we did not observe statistically significant geographic clusters on the seamount in dive 145, potentially because the seabed here was undersampled due to camera malfunction (Note the shorter length of dive 145). In the Western region, statistically significant hotspots of megafauna were observed in complex physical landscapes e.g. on top of the seamount (in dive 32) as well as on top of local abyssal hills (in dive 28). Short stretches of coldspots were located in relatively flat abyssal plains (at the start of dive 78 and at the end of dive 28). We did not observe significant outliers.

### Influence of biotic and abiotic drivers

**Fig. 8:**
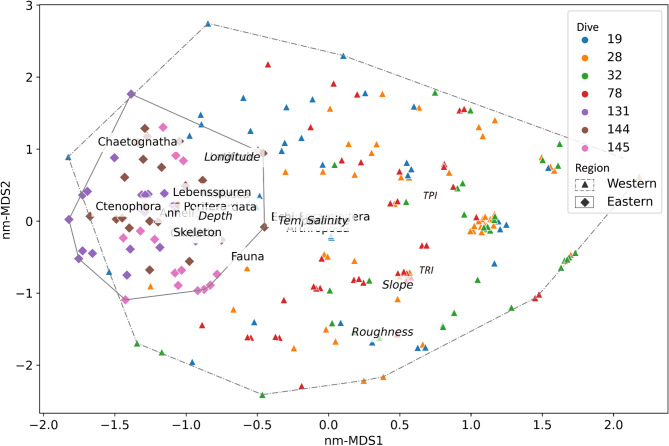
Projection of sampling units onto nm-MDS ordination space. Colour coding is based on the dives whereas the symbols distinguish between the two regions. Also superimposed in the ordination plot are taxa and environmental drivers that potentially structure megafaunal communities in the two regions. It is clear that there is a distinction between the two main megafaunal communities in the Eastern and Western region, and also that bathymetric drivers predominantly structure communities in the deeper Western region.

The ordination of all the 232 sampling units as projected onto a two-dimensional nm-MDS feature space is shown in Fig. 8. The 64 sampling units from the Eastern region mostly group together into a small tight cluster/community on the extreme left of the ordination space, whereas the remaining 168 sampling units from the Western region are scattered throughout (although they span mostly the right half of the feature space). A few smaller sub-clusters are also visible in the Western region.

In terms of biotic/community composition, the taxa that predominantly influenced the deeper Western region include Echinodermata, Foraminifera, and Arthropoda. The remaining majority of taxa predominantly influenced the shallower Eastern region, which may explain the high number of biogenic structures (Lebensspuren) that we observed in the Eastern region.

Superimposing environmental variables onto the ordination plot shows that the horizontal axis distinguishes megafaunal communities based on temperature and depth variables. This is obvious considering that the two variables map close to the boundary separating communities in the Eastern and Western regions. On the other hand, bathymetric derivatives such as slope, ruggedness, roughness and positioning index predominantly influenced megafaunal communities at great depths in the Western region.

## Discussion

So far, we have demonstrated that incorporating A.I into marine science workflows accelerates the characterisation of habitats and megafauna distribution in deep sea environments. Here, we provide further interpretations for the observed patterns, and contextualise the findings relative to other studies.

Our semi-supervised seafloor sediment classification workflow involved clustering based on encoded visual features, followed by manual interpretation of the clusters to assign semantic meaning. We chose this approach because modern implementations of the K-means clustering algorithm are fast, accurate and straightforward to use^38^, thereby enabling automated sorting of huge volumes of unlabelled images into manageable representative groupings. In conducting benthic sediment mapping for the Australian National Marine Bioregionalisation project, Lucieer et al.^39^ also point out that statistical clustering allows for more objectivity and repeatability in image-based seafloor classification when compared to manual interpretation. In another study, Diesing et al.^40^ emphasise the key role of feature space projection methods such as principal components analysis (PCA) towards enabling visual interpretability of seafloor clusters. Clustering also ensures consistency in seafloor annotation since visually similar images are almost guaranteed to be assigned the same labels, as was also pointed out by Lathrop et al. in previous benthic habitat characterisation study in New York Bight^41^. However, the clustering performance will depend on the method used to encode visual information from images: hand-engineered features like texture are best for representing obviously heterogeneous seabed e.g. as was previously used to characterise the distribution of Mn-nodules in the Clarion Clipperton Zone ^9,42^. For this study, we extracted high-level features using a pre-trained convolutional neural network that have been shown in past studies e.g. by Yamada et al.^43^ to be capable of capturing subtle variability in seabed substrate composition. Colour-coding the feature space using clustering labels produces a graphical display that allows for a quick (qualitative) visual first impression of sediment characteristics. This kind of display proves useful for decision making by marine scientists during expeditions e.g. to determine where to sample next, or to help in the choice of an appropriate image dataset for studying a given phenomenon of interest (e.g. from repositories like BIIGLE^44^ or PANGAEA^45^). We used this graphical display in feature space as our main interface for semi-automated annotation because (a) it was more convenient to assign semantic labels to clusters compared to labelling individual images, (b) it was easy to leverage contextual information e.g. characteristics of neighbouring clusters to adapt the annotation accordingly based on the underlying structure of the data, and (c) it was straightforward to detect any anomalous patterns and/or artefacts as these clusters would be unusually isolated in the feature space. In approaching seafloor classification this way, we consider automated algorithms to be useful agents for preliminary sorting, while reserving the final call to annotators with the domain expertise to resolve nuanced, granular and subtle variability in substrate composition that may be missed by algorithms.

Deploying our *FaunD-Fast* model to detect megafaunal taxa produced weak annotations that still needed to be manually inspected, refined and re-labeled, as was also done previously by Tang et al.^46^ and Zhang et al.^47^. In our case, the model was able to correctly detect instances of megafauna in images (with an accuracy of 78.1%), even though taxa labels assigned to the detections were sometimes incorrect even for organisms that were present in both the Atlantic and the Pacific. While overfitting and poor generalisation may explain the misclassification of previously unseen taxa^24^, it is not obvious why some organisms (e.g. Holothurians and Ophiuroids) that were present in both the Pacific and Atlantic were correctly detected yet misclassified, despite colour normalising the two datasets in the same way. A possible explanation is offered by Zurowietz et al.^48^ who previously pointed out that concept drift poses a big challenge for knowledge transfer across marine environments, especially in studies involving non-endemic taxa. In this context, concept drift is the phenomenon where the statistical distribution of visual properties of marine organisms shifts across datasets either gradually or suddenly, as was also highlighted by Langenkämper et al.^49^. Therefore, exactly how to develop a species detection model that generalises across oceans remains a challenging open problem that needs further investigation. In principle, such a generalizable model must be altogether agnostic to the distinct differences in terms of ecological habitats, intra- and inter-species appearance, water column properties etcetera. Recently, there have been efforts aimed at addressing these generalisation challenges by developing well-curated standardised benchmark image datasets. For example, the openly available global image database FathomNet by Katija et al.^50^ provides annotations that cover a wide range of taxa categories from different ocean environments. The goal of these benchmark datasets is to enable training and evaluation of deep learning models that would be more robust and generalizable, since the models would have been exposed to diverse visual features of marine organisms^49^. Another potential solution for poor generalisation is data augmentation, which involves the application of random geometric and photometric transformations e.g. random scaling, rotations and flipping in order to artificially increase the volume and variety of training examples, as has previously been demonstrated by Tan et al.^51^. How well these (and other) solutions work is a promising direction for further research. Despite the aforementioned challenges, our pre-trained *FaunD-Fast* model is still directly useful in situations where one only cares about binary fauna/non-fauna detections e.g. to distinguish marine organisms from other background objects in a live OFOS/ROV video feed ^52 53^.

Absolute megafaunal taxa counts that we obtained from our fauna annotation workflow required standardisation due to potential sampling bias and lack of a consistent (spatial) scale of reference. However, the choice of an optimal length (or resolution) of the sampling unit within which to standardise the annotations is not obvious but depends on the problem and ecological context, as was also previously pointed out by Enrichetti et al.^20^ and Dominguez-Carri ´et al. ^54^ In our case, we chose a fixed-size length of 100 m because we were interested in capturing localised megafaunal distribution patterns in high resolution, along linear transects whose variability in substrate characteristics was very subtle. According to guidelines from a previous study on transects and quadrats in ecology by Murray et al. ^55^, we consider our 100-metre-long sampling units to be high resolution considering that the average length of our transects was 3200 m. Murray et al. ^55^ recommended the use of high resolution sampling units whenever possible (and resource permitting), since the fine resolution allows for the capturing of granular localised distribution patterns e.g. megafauna adapted to microhabitats, specific depth gradients or substrate type over short distances. Choosing larger-sized sampling units (e.g. with resolutions of 500 m or greater) may average out small scale spatio-ecological patterns, and are best suited for providing generalised information regarding overall trends in community structure e.g. as was previously argued by Montaña et al. ^56^. In any case, we surveyed the seafloor at sufficiently high frequency (maximum 0.2 Hz), resulting in an average of 38 images within each of our 232 sampling units of each 100 m length. This sample size is sufficient for unbiased and robust biodiversity assessments using multivariate statistics, as has previously been demonstrated by Forcino et al. ^57^. Our chosen resolution was also convenient purely from a computational perspective ^58^, because visualising the 232 sampling units in the ordination feature space did not require too much memory or computing resources.

There were major differences in the distribution and abundance of megafauna between the Eastern and Western regions. As Ramos et al. also point out in their previous study of marine biodiversity off Mauritanian deep waters^59^, the regional differences in abundance may be explainable by variability in depth and geomorphological complexity of the seabed. Considering that the average depth difference between the Eastern and Western regions of our working area was approximately 700 m, the high megafaunal abundances and diversity in the shallower Eastern region might be due to food availability in the form of sinking organic matter^60^. Since the Eastern region is also relatively closer to Mauritanian shore, the region benefits more from both land-based nutrient sources as well as from nutrient enrichment from upwelling currents, as has also been previously reported by Scepanki et al.^61^. Moreover, our CTD profiles in Supplementary Figure S1 show that the shallower waters in the Eastern region also exhibit relatively warmer temperatures (2.85 °C ± 0.12) compared to the Western region (2.50 °C ± 0.08). These warmer temperatures may also have contributed to the observed high megafaunal abundance in the Eastern region, since elevated temperatures have been shown to enhance metabolic rates while also supporting a wider range of functional traits, e.g. as shown by Puerta et al.^62^ and Sweetman et al.^63^. Both the transect depth profiles and the hill-shaded bathymetric grid (Fig. 2) show the presence of structurally complex features like seamounts and local elevations in the Eastern and Western regions. In general, we observed high megafaunal abundances in these complex habitats compared to flat terrains because the complex topographies create microhabitats e.g. rocky outcrops and sediment pockets, which provide shelter and protection for megafauna while also influencing hydrodynamic conditions to create stronger currents that promote nutrient distribution and richer food webs^64^. Still, we observed higher abundances on seamounts in the Eastern region compared to those in the deeper Western region, which could be because POC flux is higher in shallow seamounts due to stronger upwelling effects, as was also previously shown by Victorero et al.^65^. Regarding the influence of environmental drivers on the observed spatial patterns, our ordination plot showed that bathymetric drivers such as slope, ruggedness, and roughness predominantly influenced megafaunal communities in the deeper Western region. This could be because habitats in the deeper seabed areas are in general more stable, with topographies that vary slowly in kilometre scale^66^. As a result, even minor variability in the bathymetric derivatives in the deeper seabed results in a more pronounced influence on hydrodynamic effects like current patterns and nutrient distribution. This is in contrast to the already complex topographies in shallower parts that naturally disrupt hydrodynamic flows, so that the effect of minor changes in bathymetric derivatives are not as pronounced e.g. as was also pointed out by Kaiser et al.^67^.

Collectively, the above findings demonstrate that incorporation of A.I into conventional image-based marine science workflows does contribute towards expedited characterisation of substrate types and megafaunal distribution as follows: First there are obvious speed gains since human effort is required to semantically label only the A.I generated annotations instead of the entire dataset. Second, our workflow practically demonstrates how projecting seafloor images onto feature space does allow for a quick at-a-glance visualisation (and interpretation) of natural benthic habitat groupings, including any anomalies that require further investigations. Third, outputs from our A.I workflows (e.g. data matrices) seamlessly integrate with existing classical spatio-ecological workflows such as ordination and spatial autocorrelation analysis. This integration allows us to automate only the necessary repetitive time-consuming tasks (like annotation), while avoiding unnecessary re-invention of the wheel in downstream analysis. Fourth, despite the occasional misclassifications, our model does generalise across oceans to the extent that it correctly detects and localises organisms in images. This is directly useful in applications such as rapid underwater video analysis to e.g. extract relevant frames for subsequent semantic labelling. Therefore, we conclude that automated image analysis workflows have the capacity to efficiently extract actionable insights from terabyte-scale seafloor imagery, which is necessary to complement both ongoing and planned development of timely marine baseline information for monitoring remote benthic ecosystems at regional and global scale.

## Methods

### Study Area

**Fig. 9:**
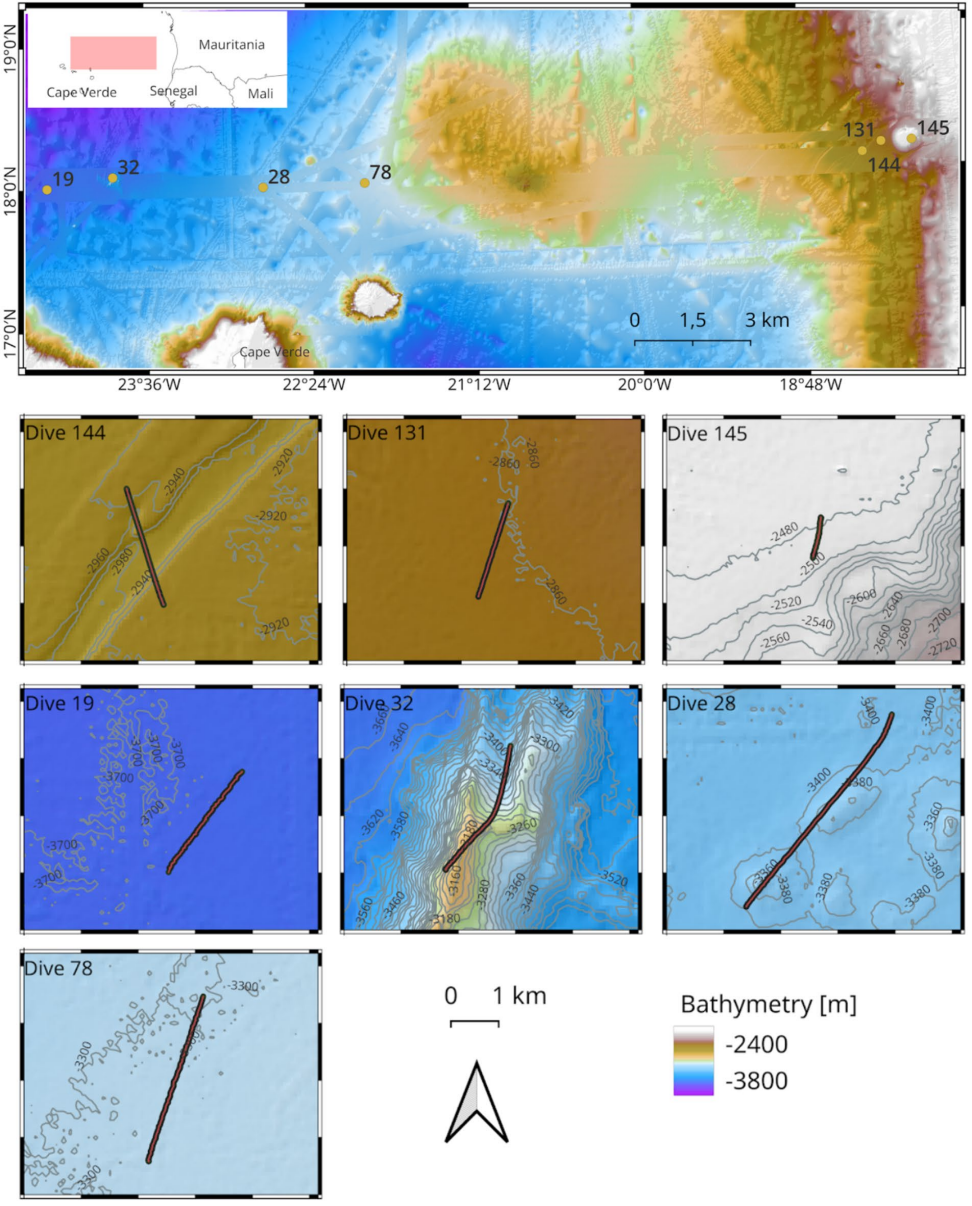
Map showing the OFOS (camera) deployment tracks during cruise M182 to the tropical North Atlantic, which we conducted on board RV Meteor between May - July 2022. Note that in this map we only show camera deployments from deep sea environments (> 2,000 m water depth). Also notice that dive 145 is shorter than the other dives because the camera malfunctioned after just 1 h 13 min of bottom time.

Our working area offshore Mauritania and North of Cape Verdes (Fig. 9) followed an East-West orientation, with a total of seven camera deployment stations distributed between the Eastern region (comprising dives 131, 144, 145) and Western region (dives 19, 32, 28, 78).

The Eastern region was shallower with depths ranging between − 2470 m up to -2970 m. This region was characterised by topographically complex features such as a seamount (in dive 145), as well as the submarine canyon at -2920 m water depth (in dive 144). The canyon exhibits steep near-vertical 20-metre-high walls with a cross section that is approximately 500 m wide, marking a visibly distinct narrow passage on the seabed. CTD profiles further show that the water masses in the Eastern region are relatively warmer, with average temperatures of (2.85 °C ± 0.12).

In contrast, the Western region was deeper and relatively colder, with depth ranges of between − 3128 m and − 3693 m, and average temperatures of (2.50 °C ± 0.08). There was also a seamount in this region that rose approximately 200 m high from the seabed (in dive 32), as well as a pair of adjacent 40-metre high locally elevated abyssal hills (in dive 28). For further details on the physical and water mass properties for respective dives, please refer to the CTD profiles in Supplementary Figure S1.

### Image dataset

We surveyed the working area between May 31st and July 10th 2022 on board RV METEOR during expedition M182^68^. The aim of the cruise was to study the influence of mesoscale eddies on (a) biogeochemical processes in the Eastern boundary upwelling systems, and (b) modulation of organic matter transport from the surface waters down to the seafloor^6^. The sampling campaign involved the deployment of several gears and systems such as the extended Ocean Floor Observation Systems (XOFOS), CTDs, MultiNets, biogeochemical landers, AUVs, and ship-based multibeam bathymetry.

For this particular study, we used still images collected by the XOFOS, which is an imaging platform comprising a topside unit on the ship (for power, data connection and live video feed), as well as a subsea unit that is lowered into the water column by a winch system to survey the seafloor (up to 6000 m deep). The subsea unit comprises a heavy metal frame that houses forward- and downward-looking 24-megapixel digital Ocean Imaging Systems camera (DSC 24000). The XOFOS records images automatically at a constant frequency that is set before deployment, as well as through hotkey functionality for recording adhoc images or random events of interest. In addition to the camera, the XOFOS is also fitted with downward facing LED lights/flashers and a USBL positioning system that tracks the platform position during image acquisition. Additional sensors such as ADCPs, CTDs and other loggers may also be attached to the XOFOS, allowing for a straightforward integration of auxiliary datasets and images based on synchronised timestamps.

Based on the above set up, we obtained 9000 still images by photographing the seabed at constant frequencies of 0.07 Hz (in dives 28, 32), 0.10 Hz (in dives 78, 131, 144, 145) and 0.2 Hz (in dive 19). Out of the 9000 acquired images, we excluded 162 images whose visual quality was severely degraded due to poor illumination conditions and water quality effects. Therefore, the optimal number of images that were subsequently used in this study were 8838.

The seven XOFOS dives covered a total track length of 22.7 km, which represents a visual footprint of approximately 73,616 m^2^ on the seafloor. We estimated this visual footprint based on the fixed opening angles of the camera (48° horizontal, 33° vertical) and the acquisition heights of respective images above the seafloor. Table 1 below provides an overview of our camera deployments, while the cruise report contains further technical details regarding the image acquisition setup^68^.

**Table 1:** Overview of camera deployments during expedition M182.

Dive	start time	end time	start lon	start lat	end lon	end lat	start depth [m]	end depth [m]	bottom time	track length [m]	visual footprint [sq. m]	sampling units	total images	annotated images
**19**	6/4/2022 0:04	6/4/2022 3:20	-24° 20.049’	18° 0.080’	-24° 19.182’	18° 1.163’	-3691	-3689	3:16:35	3758.07	16960.03	39	2360	537
**28**	6/5/2022 15:49	6/5/2022 22:00	-22° 45.410’	17° 58.856’	-22° 43.630’	18° 1.000’	-3392	-3397	6:11:01	5257.78	17141.5	53	1485	398
**32**	6/6/2022 16:35	6/6/2022 20:27	-23° 51.116’	18° 4.529’	-23° 50.317’	18° 5.921’	-3161	-3403	3:51:16	3374.81	12311.05	34	926	283
**78**	6/13/2022 23:36	6/14/2022 3:40	-22° 0.139’	17° 59.787’	-21° 59.442’	18° 1.635’	-3285	-3293	4:04:31	4148.12	11041.96	42	1468	419
**131**	6/24/2022 1:13	6/24/2022 4:02	-18° 13.275’	18° 9.872’	-18° 12.871’	18° 10.899’	-2855	-2853	2:49:40	2128.6	6591.97	22	1019	676
**144**	6/26/2022 20:18	6/26/2022 23:30	-18° 21.193’	18° 5.852’	-18° 21.563’	18° 7.174’	-2924	-2930	3:11:40	2603.81	7100.57	27	1150	1042
**145**	6/27/2022 4:06	6/27/2022 5:18	-17° 59.618’	18° 10.827’	-17° 59.735’	18° 10.380’	-2470	-2486	1:11:31	1445.7	2469.82	15	430	417

We have uploaded the entire image collection (and annotations) to GEOMAR’s annotation portal BIIGLE, which is accessible from here (https://annotate.geomar.de/projects/65*).* Supplementary Figure S6 shows an example seafloor image with annotated megafaunal taxa.

### Image visibility improvement

The visual quality of deep-sea images is usually degraded by the scattering, absorption and attenuation effects of artificial LED light as it propagates through the water during image acquisition^69^. While absorption of light by water molecules causes wavelength-dependent colour distortion, scattering by suspended particles results in images with blue/greenish haze and low contrast^70^. Moreover, the inability of the imaging platform to maintain a consistent altitude above the seafloor leads to variable scene brightness, as well as inconsistent scale among individual images^9^. It is therefore necessary to account for these distortions before extracting semantic information for use in downstream ecological analysis.

Our workflow for improving the image quality involved three main steps: First, we applied z-score normalisation to batches of chronologically-sorted images to dehaze them, and also to reduce the effect of the gradual intensity drop-off towards the image edges and corners; Second, we applied adaptive histogram equalisation to local image tiles, which resulted in improved overall contrast for the entire image; Finally, we used histogram matching (based on a manually chosen reference image) to correct for uneven scene brightness among individual images. (Please see further technical details in Mbani et al.^9^).

Regarding parameterizations, we chose to manually tune hyperparameters that require domain knowledge, so as to ensure that our downstream workflow(s) produce semantically meaningful results. For example, our visibility improvement workflow above operated on successive batches, with each batch containing 32 images. The rationale for choosing this batch size was (1) the images could reasonably fit into our computer memory, and (2) the altitude of the XOFOS is relatively constant over short distances within which distortion effects are similarly constant. Therefore, z-score normalisation over such small-sized batches effectively removes batch-specific mean effects without introducing artefacts. Colour normalising over large batch sizes is undesirable because respective batches would comprise images with significantly different radiometric properties and inconsistent biases.

Colour normalisation produces consistent and comparable images, ensuring that downstream tasks e.g. classification are based on actual semantic seafloor properties rather than arbitrary water column or radiometric properties.

### Semi-automated classification of seafloor substrate

The goal of seafloor classification is to partition the seabed into semantically meaningful habitat classes. Our (semi) automated approach involved first clustering the images into natural groupings based on automatically extracted visual features, and later inspecting the generated clusters to manually assign semantic class labels. This approach reduced the required manual annotation effort, while still directly incorporating domain expertise into the annotation process.

We used a pre-trained InceptionV3 convolutional neural network to extract high-level features from entire images, encoding the visual information into feature vectors. First, we divided the 8838 images into smaller batches, with each batch containing 32 images. Feature extraction was then done batchwise to reduce memory requirements (our GPU memory was 11 gigabytes), while simultaneously allowing for parallel processing. The output of the feature extraction process was a 2048-dimensional feature vector (for each image) that compactly encoded relevant visual information. We aggregated and stacked the feature vectors from all the batches into rows of a data matrix. K-means algorithm was then applied to this data matrix resulting in 12 distinct seafloor habitats (or clusters), with the number of classes being chosen as the maximiser of the K-means objective function after experimenting with a range of clusters between 2 and 20.

Finally, we manually inspected image sub-samples from each cluster, and interpreted them based on their contextual physical/geological characteristics as well as visible biogenic features like bioturbation, burrowed mounds and other seafloor features.

### Semi-automated annotation of megafaunal taxa

Automated workflows are required to expedite annotation of the huge volumes of underwater imagery collected during scientific expeditions. Therefore, we deployed our automated megafauna detection workflow *FaunD-Fast*^23^ to detect, localise and classify megafaunal taxa from image sequences. *FaunD-Fast* was built on top of the Tensorflow Object Detection API^71^, and is an instance of the two-stage Faster R-CNN model^31^. During training, the Faster R-CNN first uses a Region Proposal Network to scan the entire image and propose candidate regions with high likelihood of containing objects. In the second stage, features are extracted from the region proposals for subsequent classification and refinement of object bounding box coordinates relative to ground-truth (training examples)^31^.

Our training setup involved an Ubuntu 18.04 LTS operating system powered by an 11-gigabyte Geforce RTX 2080 graphics card. The bounding box coordinates that we used to train *FaunD-Fast* were for megafaunal taxa from the Clarion Clipperton Zone (our previous working area). However, we still used the model to detect and classify megafauna from our new seafloor images from the tropical North Atlantic. The goal was to quickly generate a set of weak (imprecise) annotations that we could later present to domain experts for refinement and semantic labelling. Approaching the annotation this way was more efficient compared to asking annotators to manually inspect the entire image collection outright. This annotation strategy also naturally allows for the incorporation of rich, nuanced and contextualised domain expertise into the otherwise automated workflow.

Finally, we retrained *FaunD-Fast* using semantic (Atlantic-specific) annotated examples from above, assessed the model performance using standard coco metrics, and eventually deployed the model in inference mode to detect megafauna exhaustively from the entire image collection. Our model outputs included images overlaid with bounding boxes of detected taxa (e.g. Supplementary Figure S6), as well as a detections summary table whose rows represent individual images and columns indicate absolute counts of respective taxa detected in the corresponding image (Supplementary Table S1).

### Converting absolute megafaunal counts to standardised abundances

**Fig. 10:**
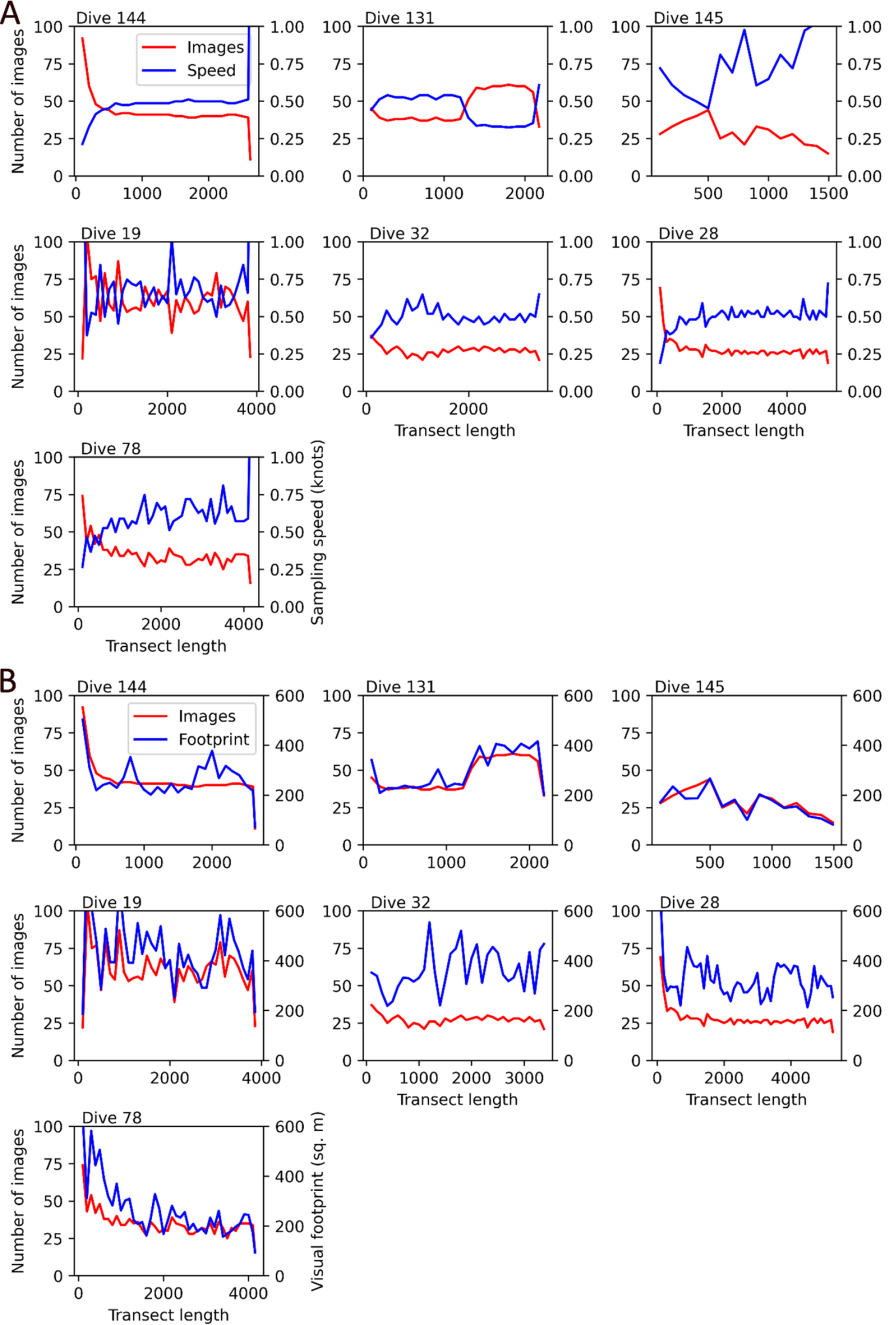
Variability in number of images, XOFOS speed, and actual observed area within fixed-size (100-metre-long) sampling units along respective survey transects. (A) shows an obvious inverse relationship between the speed of the XOFOS and the number of acquired images, whereas in (B) the relationship between number of images and observed visual footprint is not obvious, especially in topographically complex terrains like seamounts. Note that the relatively high number of images at the start of some transects is caused by the initial stabilisation phase, where the deployed XOFOS first experiences twists and turns in more or less the same location before eventually maintaining a linear transect.

As mentioned earlier, variability in camera altitude (among images) and sampling frequency (among dives) introduces biases that render image-level taxa counts incomparable. Sampling bias for example may cause certain regions (or depth ranges) of the seafloor to be either over- or under-represented e.g. as measured by the number of images per unit area, whereas scaling bias affects the size of objects as well as the visual footprint of individual images. Figure 10 illustrates the manifestation of the above biases along respective survey transects in our working area.

To define consistent sampling units for comparing spatio-ecological patterns, we partitioned each transect into successive 100-metre-long segments to fix the spatial scale (or resolution). Within each segment, we summed up annotated (absolute) taxa counts, and also calculated the total observed area by summing up the visual footprints (in square metres) for all images in the respective segment.

For a fixed sampling frequency, Fig. 10A illustrates that the lower the speed of the imaging platform the smaller the distance between successive images, resulting in a relatively high number of images per unit distance (and vice versa). Although the number of images generally correlates positively with observed seafloor area, Fig. 10B shows that visual footprint is a function of not only towing speed and the set camera altitude, but also of terrain complexity. This is evident from the high variability in visual footprint along transects that traverse a seamount (dives 32) and local abyssal hills (dive 28), despite relatively constant towing speed (or number of images). It is therefore necessary to standardise the annotated image-level taxa counts e.g. by converting them into abundances per unit area.

Therefore, we generated abundances for each sampling unit by dividing absolute taxa counts by the total observed area. The results were aggregated into an abundance data matrix, whose rows correspond to sampling units (instead of individual images), while columns represent relative abundances of respective taxa (see Supplementary Table S2).

### Assessing the spatial distribution of megafauna

Characterising the spatial distribution of megabenthic fauna allows us to provide geographic context to the image-derived abundances. Here, we used the centroid coordinates of each sampling unit to plot their locations in map view. We applied quantile classification to bin abundances into eight distinct classes that we used to colour-code the choropleth maps (Fig. 6). This visual representation allowed for a straightforward interpretation of the variability in megafaunal abundances relative to the background bathymetry that we plotted as a basemap. In addition to the planar map view, we also plotted the abundances along elevation profiles of each transect, to investigate variability at local heights.

To complement the qualitative choropleth mapping, we used quantitative measures of spatial autocorrelation to reveal regions of the seafloor where geographic clustering of megafauna was statistically significant (beyond what would be expected from random chance). In this context, spatial autocorrelation quantifies the degree to which the abundance of megafauna in a given sampling unit is similar to the average abundances of neighbouring sampling units. Thus, the choice of the optimal neighbourhood size is key because it directly influences the outcome and subsequent interpretation of hotspot analysis results: overly large neighbourhood sizes may smooth away local spatial patterns, whereas overly small neighbourhoods may be very sensitive to noise and other spurious artefacts in the abundance data matrix. Here, we defined our optimal neighbourhood size to comprise six nearest neighbours, after empirically observing that for most dives, the rate change in spatial autocorrelation (Moran’s I) does not change significantly from around the sixth-order neighbourhood. (Supplementary Figure S7).

To detect megafaunal hotspots and coldspots, we first used k-nearest neighbour algorithm^72^ to construct a graph that connects each sampling unit to its six nearest neighbours (based on geographic proximity). Based on this neighbourhood graph, we calculated Local Indicators of Spatial Association (LISA) statistics for each sampling unit, which identified localised regions where megafaunal abundances were significantly higher or lower than would be expected from spatial randomness. To classify these geographic clusters (as either hotspots or coldspots), we projected the LISA statistics onto a Moran’s scatterplot (Supplementary Figure S8), which shows the relationship between the abundance of each sampling unit versus the average abundances of its neighbours (spatial lag). Depending on where a given sampling unit was located on this scatterplot, we classified it as either a hotspot (high-high abundances), coldspot (low-low abundances) or an outlier (low-high or high-low abundances). Finally, we assessed the statistical significance of the observed spatial patterns (or LISA statistics) by conducting a randomised hypothesis test under the null hypothesis of complete spatial randomness.

### Assessing megafaunal biodiversity and community composition

Benthic biodiversity assessments are key towards understanding the overall ecosystem health and functionality. Here, we used standard deviation of megafaunal abundances and Shannon diversity index to measure diversity in both the Eastern and Western region. This regional comparison of diversity allowed us to simultaneously assess both spatial variability and depth-wise zonation patterns of megafauna, since the Eastern and Western regions vary by water depth and distance to shore (with distinct differences in carbon export to the seafloor, upwelling processes, and input of terrigenous material).

We identified clusters of megafaunal communities using non-parametric multivariate statistics. First, we applied double-root transformation to the abundance data matrix to stabilise the variance and moderate the influence of dominant taxa (abundance data typically contains many low values and few high values). Next, we used the transformed abundances to generate a Bray-Curtis similarity matrix that captures the degree of biotic (dis)similarity among the sampling units. We then applied hierarchical agglomerative clustering (with group-average linking) to this similarity matrix, thereby revealing clusters of sampling units with similar biotic composition.

To formally test whether the differences among the major clusters of megafaunal communities was statistically significant, we performed an analysis of similarity (ANOSIM). ANOSIM calculates a test statistic *R* that captures the average difference between inter- and intra-community similarities, with the null hypothesis *H0* defined as: *There is no significant difference in biotic composition among the main megafaunal communities*. In addition, we used a similarity percentages analysis (SIMPER) to identify taxa that contributed the most towards the separation among respective clusters of megafaunal communities.

Finally, we visualised the inter-relationships among megafaunal communities by projecting the sampling units onto a two-dimensional ordination (feature) space using non-metric multidimensional scaling (nm-MDS). We also superimposed onto the ordination plot taxa and environmental variables that we sampled at the centroids of respective sampling units. These abiotic variables included: depth, slope, topographic position index, terrain ruggedness, salinity, temperature and longitude. This graphical representation allows for a convenient visual interpretation of the association between biotic and abiotic variables, together with their influence on the identified megafaunal communities. (Note that we omitted latitude since all our deployments were along an East-West transect. Also, longitude here is proportional to distance from shore but not to depth, even though the two variables are correlated to some extent).

## Data Availability

The datasets presented in this study can be found online in BIIGLE here https://annotate.geomar.de/projects/65. Intermediate data generated during the analysis is also provided in the supplementary materials as an excel file. To request the data used in this study, please contact Prof. Dr. Jens Greinert (jgreinert@geomar.de).
